# Molecular characterization and antibiotic resistance of enterotoxigenic and entero-aggregative *Escherichia coli* isolated from raw milk and unpasteurized cheeses

**Published:** 2014

**Authors:** Mojtaba Bonyadian, Hamdallah Moshtaghi, Mariam Akhavan Taheri

**Affiliations:** 1 *Department of Health and Food Quality Control, Faculty of Veterinary Medicine, Shahrekord University, Shahrekord, Iran;*; 2 * Graduated in Veterinary Medicine, Faculty of Veterinary Medicine, Shahrekord University, Shahrekord, Iran.*

**Keywords:** Antibiotic resistance, Raw milk, Toxigenic *E. coli*, Unpasteurized cheese

## Abstract

The aim of this study was to determine the occurrence of enterotoxigenic and enteroaggregative *Escherichia coli* strains and antibiotic resistance of the isolates in raw milk and unpasteurized cheese. Out of 200 samples of raw milk and 50 samples of unpasteurized cheeses, 96 and 24 strains of *E. coli* were isolated, respectively. Polymerase chain reaction (PCR) was used to detect the genes encoding heat-stable enterotoxin a (STa), heat-stable enterotoxin b (STb), heat labile toxin (LT) and enteroaggregative heat-stable toxin1 (EAST1). Twelve out of 120 (10.00%) isolates harbored the gene for EAST1, 2(1.66%) isolates were detected as producing STb and LT toxins and 12 (10.00%) strains contained STb and EAST1 genes. None of the strains contain the STa gene. All of the strains were tested for antibiotic resistance by disk diffusion method. Disks included: ciprofloxacin (CFN), trimetoprim-sulfamethoxazole (TSX), oxytetracycline (OTC), gentamicin (GMN), cephalexin (CPN), nalidixic acid (NDA) and nitrofurantoin (NFN), ampicillin (AMP), neomycin (NEO) and streptomycin (STM). Among 120 isolated strains of *E. coli*, the resistance to each antibiotics were as follows: OTC100%, CPN 86.00%, NDA 56.00%, NFN 42.00%, GMN 30.00%, TSX 28.00%, CFN 20%, AM 23.40% and STM 4.25%. None of the isolates were resistant to NEO. The present data indicate that different resistant *E. coli* pathogens may be found in raw milk and unpasteurized cheese. It poses an infection risk for human and transferring the resistant factors to microflora of the consumers gut.

## Introduction

*Escherichia coli *is generally considered as a commensal member of the normal intestinal micro flora in people and animals. Pathogenic *E. coli *can cause intestinal and extra-intestinal infections in mammalian and avian hosts.^1^ At present, several classes of enterovirulent *E. coli*, namely enterotoxigenic *E. coli *(ETEC), enteropathogenic *E. coli* (EPEC), enterohaemorrhagic *E. coli *(EHEC), enteroinvasive *E. coli *(EIEC), enteroaggregative *E. coli* (EAEC), diarrhoea-associated hemolytic *E. coli *and cytolethal distending toxin (CLDT)-producing have been recognized.^2^ Ingestion of contaminated water or food results in ETEC infection causing watery diarrhea, nausea, abdominal cramps and a low-grade fever.^3^ Two enterotoxins, heat labile toxin (LT) and heat-stable (ST) play a distinct role in the pathogenesis of enterotoxigenic straines.^4^ The LT is inactivated when exposed to 60 ˚C for 15 min.^5^ The genes encoding LT (*elt or etx*) reside on a plasmid which may also harbor genes encoding ST and/or colonization factor antigen (CFA).^6^ There are two classes of heat-stable toxins, STa and STb, which differ structurally and functionally. They are small, monomeric toxins resistant to 15 min of heat treatment at 100 ˚C. The genes encoding both STa and STb toxins are present on plasmids.^2^^-^^5^

Enteroaggrigative *E. coli* (EAEC) strains are associated with acute or persistent diarrhea among children in tropical and nontropical temperate regions; and have been implicated in food-borne outbreaks, nosocomial infections and travelers' diarrhea.^7^^-^^8^ Enteroaggregative heat-stable toxin 1 (EAST1), which was first identified in human isolates of EAEC, is a 4.10 kDa peptide sharing 50.00% homology with STa.^9^ It has been proposed that the mechanism of action of EAST1 is similar to STa in increasing cyclic goanidin monophosphate (cGMP), however, the exact role of EAST1 in the development of diarrhea is still unclear.^2^^-^^5^ DNA probes and PCR assays have been used frequently for the detection of ST and LT genes along with other virulence factors of ETEC strains.^10^ Recently large outbreak of bloody diarrhoea complicated by hemolytic uremic syndrome (HUS) has been observed in the north of Germany. WHO and German authorities confirmed that this epidemic was related to infection by new, unusual enteroaggregative Shiga toxin/verotoxin-producing *E. coli* 014:H4 strain.^11^^-^^12^

The spread of antibiotic-resistant bacteria in the environment is dependent on the presence and transfer of resistance genes among microorganisms, mutations, and selection pressure to keep these genes in a population. Selection pressure has been neatly provided by the approximately 50 million pounds of antibiotics that are produced and used each year in the United States.^13^ Only half of these antibiotics are used for humans, while the remainder are administered to animals or other organisms.^14^ The causes and effects of antibiotic-overuse are varied. One of the most controversial applications of promotion in livestock, and this application has raised concerns about its contribution to the presence of resistant bacteria in humans.^15^^-^^16^

The objective of the present study was to investigate the incidence of ETEC and EAEC in raw milks and unpasteurized cheeses and identify the virulence genes and antibiotic resistance of the isolates. 

## Materials and Methods

**Bacterial strains and growth conditions.** In this study 200 samples of raw milk from bulk of the dairy farms and 50 samples of unpasteurized soft feta cheeses from retail shops were analyzed for detection of *E. coli *strains. After preparing the 1:10 dilution, samples were streaked onto Mac Conkey agar (Merck, Darmstadt, Germany) plates and incubated at 37 ˚C for 24 hr. Five red colonies showing *E. coli* characteristics were submitted to Gram staining and identified by standard biochemical tests: oxidase, indole, Simon’s citrate, urease and hydrogen sulfide as previously described.^17^

**PCR primers and amplification. **One hundred and twenty strains of *E. coli* were isolated from raw milk and unpasteurized cheeses. Simplex PCR assays were used to detect the presence of the genes encoding STa, STb, LT and EAST1 toxins by using specific primers (CinnaGen, Tehran, Iran). The culture of each isolates was prepared by inoculating in tryptose soy broth (Merck Darmstadt, Germany) and incubated at 37 ˚C for 24 hr. An aliquot was diluted in 450 µL of distilled water and boiled for 10 min. Then it was centrifuged (Seward, London, UK) at 3000 *g* for 2 min and supernatant was taken as DNA template.^18^ The PCR was carried out in a final reaction volume of 23 μL using 0.2 mL thin wall PCR tube. A master mix (25 mL PCR buffer 10X, 10 mL MgCl_2_, 7.5 of deoxy-nucleotide triphosphates and 1.5 mL of *Taq *DNA polymerase) for minimum of 10 samples were prepared and dispersed into PCR tubes and 2.0 μL sample of DNA was added into each tube to make the final volume of 23 μL. PCR tubes containing the mixture were tapped gently and quickly spun at 1000 *g* for few seconds. The tubes were transferred to thermal cycler (Biorad, California, USA) and the thermal cycle was done for 35 cycles as follows: denaturation 94 ˚C for 1 min, annealing variable (Table 1),^18^ extension 72 ˚C for 1 min.

**Antimicrobial susceptibility testing. **Antimicrobial susceptibility testing was carried out by the disk diffusion method according to the recommendations by the National Committee for Clinical Laboratory Standards (NCCLS).^19^ As recommended by the NCCLS, Mueller-Hinton agar (Merck, Darmstadt, Germany) batches were used as culture medium. The antimicrobial agent discs were obtained from CinnaGen (Tehran, Iran). The isolates were tested against commonly used antibiotics such as: CFN, TSX, OTC, GMN, CPN, NDA, NFN, AMP, NEO and STM. The zone diameters around all disks were interpreted using the recommendations of the Clinical and Laboratory Standards Institute (CLSI).

**Table 1:** Characterization of primers using in this study

**Primers**	**Sequences (5’- 3’)**	**Size of amplified product (bp)**	**Annealing temperature (**˚C**)**	**Positive control**
***LT*** **-F**	TTA CGG CGT TAC TAT CCT CTC TA	275	60	P97- 2554B
***LT*** **-R**	GGT CTC GGT CAG ATA TGT GAT TC	275	60	O149:K91
***STa*** **-F**	TCC CCT CTT TTA GTC AGT CAA CTG	163	60	P97-2554B
***STa*** **-R**	GCA CAG GCA GGA TTA CAA CAA AGT	163	60	O146:K91
***STb*** **-F**	GCA ATA AGG TTG AGG TGA T	368	60	P97-2554B
***STb*** **-R**	GCC TGC AGT GAG AAA TGG AC	368	60	0149:K91
***EAST1-*** **F ** ***E***	TCG GAT GCC ATC AAC ACA GT	125	55	P97-2554B
***AST1*** **-R**	GTC GCG AGT GAC GGC TTT GTA G	125	55	0149:K91

## Results

**Fig. 1:**
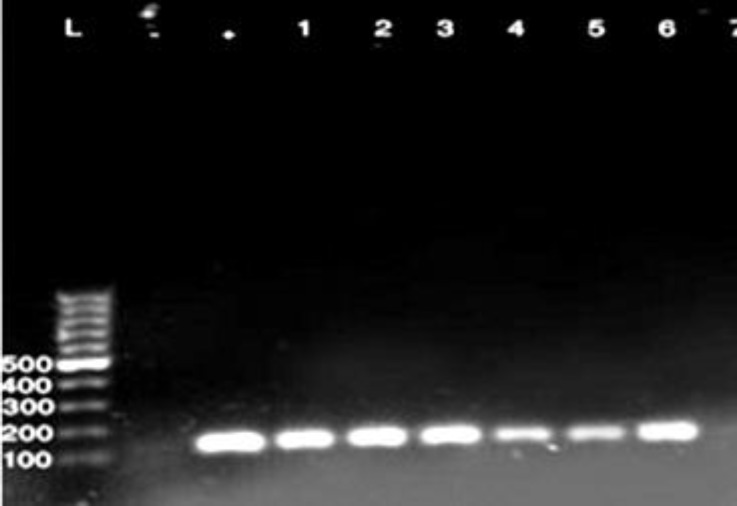
PCR detection of EAST1 gene of *E. coli*, (125 bp). **L:** Ladder, **+** : Positive control, and **-**: Negative control

**Fig. 2:**
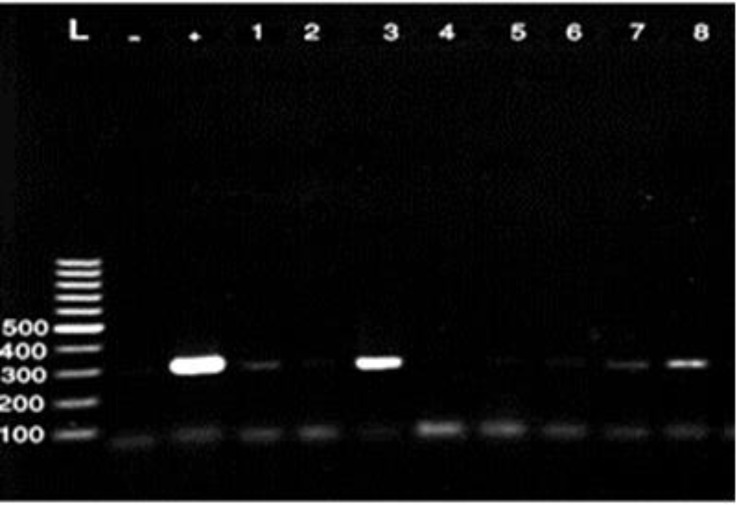
PCR detection of LT gene of *E. coli*, (275 bp). **L:** Ladder, **+**: Positive control, and **-**: Negative control

**Fig. 3:**
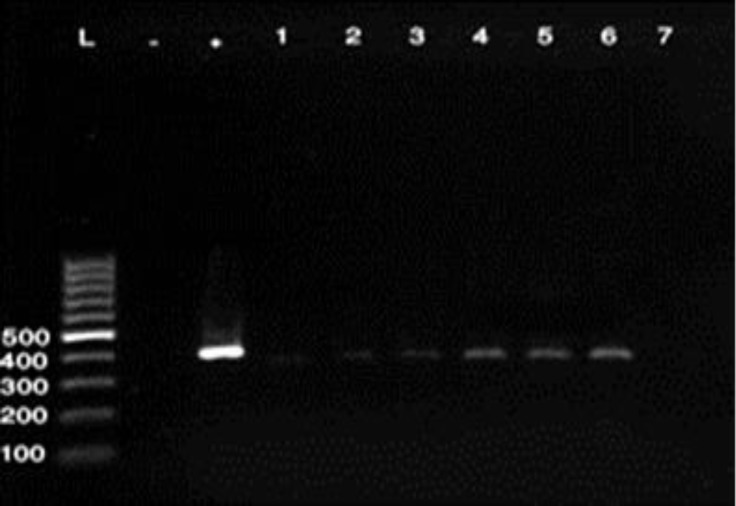
PCR detection of STb gene of *E. coli*, (368 bp). **L:** Ladder, **+**: Positive control, and **-**: Negative control

One hundred twenty isolates of *E. coli* strains from 200 samples of raw milk and 50 samples of unpasteurized chesses were submitted to PCR for detection of four virulence genes. Out of 120 investigated strains from different samples a total of 26 potentially virulent strains identified (21.66%), (Table 2). Regarding to the results of PCR tests 12 out of 120 (10.00%) isolates harbored the gene for EAST1, 2(1.66%) LT and STb and 12(10.00%) contain both STb and EAST1 genes. None of the strains harbored the STa gene, (Table 2 and Figs. 1, 2 and 3).

All (100%) of 120 strains were resistant to OTC. The rates of resistance were CFN 20.00%, TSX 28.00%, GMN 30.00%, CPN 86.00%, NDA 36.00%, NFN 42.00%, AMP 23.40% and STM 4.25%. None of the isolates were resistant to NEO, (Table 3). Some isolates demonstrated a zone of inhibition at the limit (the zone between resistance and susceptibility). To facilitate our analysis, these isolates were considered resistant. 

In this study, the results showed that out of 12 strains containing both STb and EAST1 genes 6(50.00%) strains were resistant to NFN, NDA, CPN, OTC and TSX. Out of 12strains containing EAST1 gene 4 (30.00%) were resistant to OTC, TSX, CPN and NFN. Two strain containing both LT and STb genes were resistant to OTC, TSX, CPN, GMN and NFN. 

**Table 2:** Incidence of virulence genes in *E.* *coli* isolated from raw milk and unpasteurized cheese

**Samples**	**No. of isolates**	**EAST1**	**STb and EAST1**	**LT& STb**	**STa**
**Milk**	96	10(13.88%)	12(16.60%)	2(2.77%)	0
**cheese**	24	2(7.14%)	0	0	0
**Total**	120	12(10.00%)	12(10.00%)	2(1.66%)	0

**Table 3:** Resistance of *E.* *coli* isolated from raw milk and unpasteurized cheese, as determined by disk diffusion method

**Samples**	**Number (Percentage) of resistant ** ***E. coli *** **isolates against antimicrobials** *									
	**CFN**	**NFN **	**NDA **	**CPN **	**GMN**	**OTC **	**TSX **	**AMP **	**STP **	**NEO**
**Milk**	16 (16.66)	29(30.55)	21(22.22)	77(80.55)	29(30.55)	96(100)	21(22.22)	33(34)	1(1.04)	0(0)
**cheese**	7(28.5)	17(71.4)	17(71.4)	24(100)	7(28.5)	24(100)	10(42.85)	3(14)	1(4)	0(0)
**Total**	24(20)	50(42)	43(36)	103(86)	36(30)	120(100)	34(28)	28(23.4)	5(4.25)	0(0)

* Ciprofloxacin (CFN), Nitrofurantoin (NFN), Nalidixic acid (NDA), Cephalexin (CPN), Gentamicin (GMN), Oxytetracycline (OTC), Trimetoprim-Sulfamethoxazole (TSX), Ampicillin (AMP), Streptomycin (STM), Neomycin (NEO).

## Discussion

Toxigenic *E. coli* is the most common bacterial agent of diarrhea in human and animals in developing countries.^20^ Treatment of enteric *E. coli* infection include the use of antimicrobials but increasing resistance to first-line of antimicrobial causes problems for human and animal .^21^^-^^23^ The antimicrobial resistance may be as a result of inappropriate and wide use of different antibiotics to treat infection. Resistance to currently used antimicrobial agents among enteric pathogens has increased dramatically worldwide during the past decade.^24^^-^^26^ In developing countries, TSX, AMP and tetracycline (TCN) are widely used in human to treat diarrhea because of their low cost and availability.^27^ The widespread use of these antibiotics has resulted in an increased prevalence of resistance to these antibiotics by diarrheagenic bacteria, thereby raising concern among veterinarian and general practitioners and pediatricians, especially in developing countries.^28^

This study revealed that 21.66% of the* E. coli *strains isolated from raw milk and unpasteurized cheeses contained EAST1, STb and LT genes. Toxigenic *E. coli* was identified in other foods like chicken carcasses. The prevalence of *E. coli* in chicken carcasses was 57.27% (63 of 110). Six out of 63(9.52%) of the isolates harbored the gene for LT, 1(1.58%) STb, 21(33.30%) EAST1 and 8(12.69%) contain both LT and EAST1 genes. None of the strains contained the STa genes.^29^ The results of this study showed that the resistance to antibiotic tested were as follows: CFN 20.00%, TSX 28.00%, OTC 100%, GMN 30.00%, CPN 86.00%, NDA 36.00% and NFN 42.00%. 

Studies in Vietnam revealed 86.40%, 77.20% and 19.10% of *E. coli* isolates were resistant to AMP, chloramphenicol (CMP) and TSX, respectively,^27^ whereas in Egypt the occurrence of antibiotic resistance among *E. coli* isolates from patients with acute diarrhea was 68.20%, 57.20% and 24.20% for AMP, TSX and AMP-sulbactam, respectively.^30^

In addition, a report from Iran cited by the world health organization indicated that TSX, TCN and CMP were the least effective antibiotics since 112(80.00%), 90(64.30%) and 78(55.70%) of the diarrheagenic *E. coli* isolates were resistant to these antibiotics, respectively.^20^

Several studies have determined that multi-drug resistance is common among *E. coli* isolates, especially to AMP, TSX and TCN.^31^^-^^32^ The same results have observed among the isolates of *E. coli* from animals.^33^^-^^35^ Also the resistance to the same antibiotics were observed among the *E. coli* isolated from food animal origins. Paneto *et al.* studied the occurrence of toxigenic *E. coli *in raw milk and cheese in Brazil and* E. coli *were recovered from 48 (96.00%) of the samples.^36^Three (6.00%) and 1(2.00%) of *E. coli *isolates were VTEC and ETEC, respectively. Most frequent resistance was observed to the following antimicrobials: cephalothin (60.00%), NDA (40.00%), doxycyclin (33.00%), TCN (31.00%) and AMP (29.00%). Hariharan *et al*. evaluated *in vitro* resistance to eight antimicrobials among enterotoxigenic *E. coli* from piglets and calves over a period of 13 years.^37^ The percentages of resistance of the bovine isolates in ascending order in the first eight years were ceftiofur (4.00%), gentamicin (6.00%), spectinomycin (44.00%), trimethoprim-sulphonamide (46.00%), NEO (64.00%) and OTC (81.00%). 

Cook *et al*. reported that in general, resistance to individual antimicrobials was observed more frequently in *E. coli* isolates from milk than in isolates from beef.^38^

This study showed high multidrug antibiotic resistance among toxigenic *E. coli* isolated from raw milk and unpasteurized cheese to the antibiotics frequently used to treat diarrhea in human and animal cases in Iran. 

The changing patterns of resistance to common antimicrobial agents in Iran indicates that designing a surveillance system for antimicrobial resistance and the introduction of integrated guidelines for the appropriate use of antibiotics are urgently needed. The result of this study suggests antimicrobial resistance is widespread among potentially diarrheagenic *E. coli* strains.

In this study, evaluation of antibiograms of *E. coli *strains both having virulence genes and those not having revealed that there was a relation between the presence of virulence genes and antibiotic resistance in resistant strains. The results of our study are supported by previous studies indicating that *E. coli *virulence factors could be the reason for resistance to different antibiotics.^39^^-^^40^

It can be concluded that emergence and dissemination of antimicrobial resistance in *E. coli *strains containing virulence factors may complicate treatment of certain urinary tract and enteric infections in animals. Additional research regarding the virulence markers present in *E. coli *strains from humans in Iran is certainly needed to elucidate the importance of the molecular features of potentially virulent *E. coli* strains revealed from our results in causing disease based on strain characteristics from *E. coli* isolates from human patients. Continued surveillance of *E. coli* collected from animals, foods and clinical settings, is merited to identify emerging antimicrobial-resistant phenotypes.
